# β-Ocimene, a Key Floral and Foliar Volatile Involved in Multiple Interactions between Plants and Other Organisms

**DOI:** 10.3390/molecules22071148

**Published:** 2017-07-13

**Authors:** Gerard Farré-Armengol, Iolanda Filella, Joan Llusià, Josep Peñuelas

**Affiliations:** 1 CSIC, Global Ecology Unit CREAF-CSIC-UAB, Bellaterra, 08193 Barcelona, Catalonia, Spain; iola@creaf.uab.es (I.F.); j.llusia@creaf.uab.es (J.L.); josep.penuelas@uab.cat (J.P.); 2 CREAF, Cerdanyola del Vallès, 08193 Barcelona, Catalonia, Spain; 3 Department of Ecology and Evolution, University of Salzburg, Hellbrunnerstraße 34, 5020 Salzburg, Austria

**Keywords:** *trans*-β-ocimene, (*E*)-β-ocimene, floral scent, dominant VOCs

## Abstract

β-Ocimene is a very common plant volatile released in important amounts from the leaves and flowers of many plant species. This acyclic monoterpene can play several biological functions in plants, by potentially affecting floral visitors and also by mediating defensive responses to herbivory. The ubiquity and high relative abundance of β-ocimene in the floral scents of species from most plant families and from different pollination syndromes (ranging from generalism to specialism) strongly suggest that this terpenoid may play an important role in the attraction of pollinators to flowers. We compiled abundant evidence from published studies that supports β-ocimene as a generalist attractant of a wide spectrum of pollinators. We found no studies testing behavioural responses of pollinators to β-ocimene, that could directly demonstrate or deny the function of β-ocimene in pollinator attraction; but several case studies support that the emissions of β-ocimene in flowers of different species follow marked temporal and spatial patterns of emission, which are typical from floral volatile organic compound (VOC) emissions that are involved in pollinator attraction. Furthermore, important β-ocimene emissions are induced from vegetative plant tissues after herbivory in many species, which have relevant functions in the establishment of tritrophic interactions. We thus conclude that β-ocimene is a key plant volatile with multiple relevant functions in plants, depending on the organ and the time of emission. Experimental behavioural studies on pure β-ocimene conducted with pollinating insects will be necessary to prove the assumptions made here.

## 1. Introduction

More than 1700 volatile organic compounds (VOCs) have been identified in the floral scents of flowering plants [1]. The richness of VOCs emitted by flowers is certainly very high. Floral VOCs, however, are not equally distributed across the phylogeny of flowering plants, so that the commonness and predominance of these compounds in floral scents varies widely among species. Common floral VOCs have a widespread phylogenetic distribution, which means that they are present in the floral scents of many species from different plant families. Plants generally synthesize and emit species-specific floral VOC mixtures to attract pollinators by mixing several of these common VOCs [2]. These specific floral VOC mixtures attract specialist pollinators that have evolved an innate preference for them [3] but also provide generalist pollinators with a reliable signal to effectively forage on their floral resources [4]. Some less common floral VOCs are only present in plants that are pollinated by specific pollinator groups with specific innate preferences for those VOCs [5,6,7]. Some of the rarest floral VOCs can constitute very specific and reliable communication channels between specialist pollinators and their host plants, even when they are emitted within a mixture that includes more common VOCs [3,8].

Most floral VOCs range from being relatively common to rare, but some are very common in the floral scents of species from all plant families. These volatiles, which are essentially obligate components of any floral scent, have great potential for attracting pollinators. The commonest floral VOCs, namely benzaldehyde, limonene, β-ocimene and linalool, are not only very common but are also predominant components of the floral scents of many species [9]. They belong to the two most prominent chemical groups of plant VOCs, the terpenoids and the benzenoids [10,11].

Here we review the current information on the importance of β-ocimene in plants, focusing especially on its biological functions and its ubiquitous presence in floral scents. We compiled abundant evidence from published studies supporting that β-ocimene may play a very relevant function as a general pollinator attractant. We further emphasize that β-ocimene may not only mediate pollinator attraction to flowers but plays several biological functions in plants, which vary depending on the organ and the time of emission.

## 2. Biosynthesis of β-Ocimene

**Figure 1:**
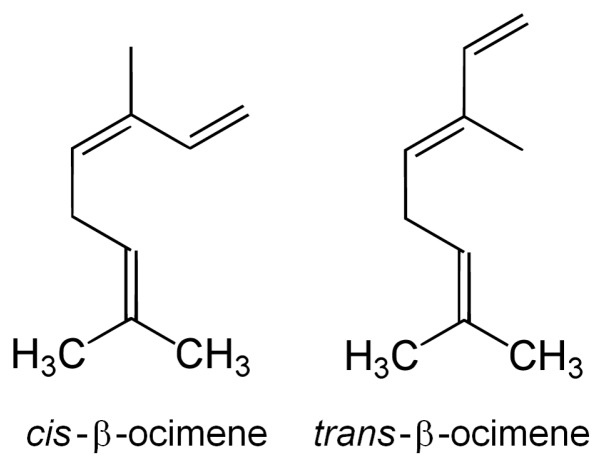
Chemical structure of the two β-ocimene stereoisomers, *cis*- and *trans*-β-ocimene (also referred to as (*Z*)- and (*E*)-β-ocimene, respectively).

β-Ocimene (3,7-dimethyl-1,3,6-octatriene) is a monoterpenoid with the chemical formula C_10_H_16_. It has two stereoisomers, *cis*- and *trans*-β-ocimene (or (*Z*)- and (*E*)-β-ocimene, respectively), which are the *cis* and *trans* forms of the central double bond (Figure 1). The *trans* isomer is more common and more abundantly emitted in floral scents than the *cis* isomer [1,9]. It is synthesized from the precursors isopentenyl pyrophosphate (IPP) and dimethylallyl pyrophosphate (DMAPP) via the methyl-erythritol-phosphate (MEP) pathway. Monoterpenes are obtained by the transformation of DMAPP and IPP into geranyl diphosphate (GPP) inside the chloroplasts, and the later synthesis of terpene compounds from GPP by enzymes called terpene synthases (TPS), which are very diversified across the phylogeny of the plant kingdom [10,12,13,14,15]. β-Ocimene is synthesized by the enzyme (*E*)-β-ocimene synthase (with the systematic name geranyl-diphosphate diphosphate-lyase ((*E*)-β-ocimene-forming)), which transforms GPP into *trans*-β-ocimene and diphosphate. (*E*)-β-Ocimene synthase produces mostly *trans*-β-ocimene (94–97%), but other monoterpenes such as *cis*-β-ocimene or myrcene are produced in low proportions (2–4% and 1–2%, respectively) [16,17,18,19]. *Cis*-β-ocimene is thus less common than *trans*-β-ocimene in floral scents; it is produced and emitted in small but detectable amounts only in species where *trans*-β-ocimene is produced in moderately high amounts.

## 3. Functions of β-Ocimene in Flowers

**Figure 2:**
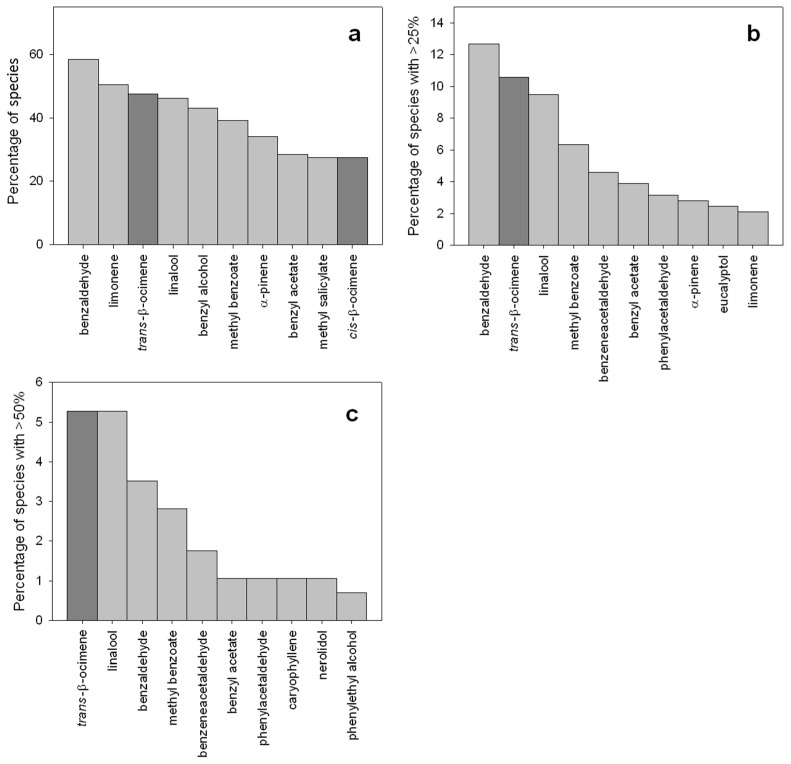
Occurrence and abundance of β-ocimene in floral scents. Bar charts showing (**a**) the percentage of plant species with the most common floral volatiles; (**b**) the percentage of plant species where the most abundant floral volatiles represent more than 25% of the total floral scent; and (**c**) the percentage of plant species where the most abundant floral volatiles represent more than 50% of the total floral scent.

**Figure 3:**
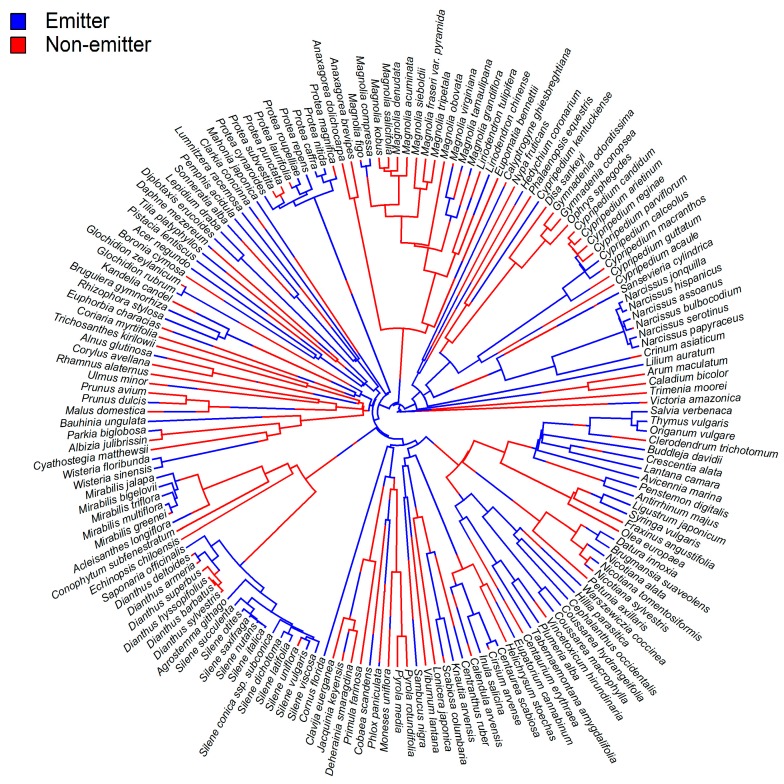
Ancestral phylogenetic reconstruction of floral emissions of *trans*-β-ocimene. One thousand trees were simulated with a mapped discrete character with states “Emitter” and “Non-emitter”. Trees had an average of 231.716 changes between states. The changes were: 117.436 Emitter→Non-emitter and 114.28 Non-emitter→Emitter. Mean times spent in each state were: 50.47% Emitter and 49.53% Non-emitter.

Ocimene is likely to play major ecological roles in flowers due to its commonness and abundance in floral scents. *Trans*-β-ocimene occurs in the floral scents of 71% of the 90 plant families in the list of identified floral-scent compounds compiled by Knudsen et al. [1]. In our recent metaanalysis of floral scents [9], *trans*-β-ocimene occurred in the floral scents of 47.5% of the 291 plant species (Figure 2) and in 75% of the 63 plant families represented. The presence of this compound in nearly a half of the species that were considered in our study may seem not too much at first glance, but when we look at the other compounds we may see that few compounds present similar levels of occurrence and predominance in floral scents (Figure 2). The ability to emit β-ocimene from flowers is widely distributed and has appeared and disappeared several times across the phylogeny of flowering plants (Figure 3). It is abundantly emitted by a wide range of plants that are pollinated by various groups of pollinators, including bees [20,21], beetles [22,23], butterflies and moths [22,24,25]. Filella et al. [26] proposed that β-ocimene was a generalist pollinator attractant, arguing that β-ocimene was emitted in high proportions by the generalist species *Muscari neglectum*, *Ranunculus gramineus*, *Euphorbia flavicoma* and *Iris lutescens*. These four species were rare at the study site in a Mediterranean shrubland community and were submitted to strong competition for pollinator visitation by the dominant plant species *Rosmarinus officinalis* and *Thymus vulgaris*. The authors also argued that all rare species that co-flowered and had to compete with *R. officinalis* and *T. vulgaris* emitted floral scents dominated by β-ocimene, because this volatile compound may help them to effectively attract a wide array of pollinators and thus to compensate for the plants' low abundance. Alternatively, the dominant species, *R. officinalis* and *T. vulgaris*, may emit more complex floral scents, which are very rich in other terpene compounds [26]. Attracting specialized pollinators that transfer pollen only from the same plant species provides several advantages for plants, compared to relying on a generalized pollination [27]. We could thus hypothesize that several plant species growing as a community reassemble a large community of pollinators by producing a global flower signal, while minor VOCs or other traits could then insure specificity in the flowers visited by pollinators.

Multiple evidence supports the role of β-ocimene in pollinator attraction in addition to its high occurrence in floral scents. β-Ocimene can effectively attract honeybees and bumblebees [28,29]. Animal-pollinated flowers have evolved mechanisms to regulate VOC emissions and make them follow particular spatial and temporal patterns of emission in order to maximize pollinator attraction and pollination success [14,30]. Spatial patterns of emission along petals or in particular organs of the flower can resemble visual nectar guides and constitute reliable guides for pollinators to find and reach the nectaries, while normally ensuring pollinator contact with the pollen and the stigmas [31]. Circadian rhythms that make floral scents follow diurnal or nocturnal periods of emission are adapted to match the periods of activity of the respective pollinators [32,33,34,35,36,37,38,39]. Ontogenical changes in floral scent along flower lifespan may also occur because flowers modify or reduce their floral scent once they are pollinated to reduce the costs, to prevent visits by pollinators and other floral visitors that can have harmful effects on floral structures, and to direct pollinator visits to unpollinated flowers [37,40,41,42]. There are several case studies demonstrating that β-ocimene emissions of different species show patterns of emission similar to those mentioned. *Ranunculus acris* flowers have a floral scent dominated by β-ocimene and its petals present emissions that differ quantitatively between the apical and basal regions, paralleling optical nectar-guide patterns that guide pollinators to the nectaries [31]. The flowers of *Mirabilis jalapa* emit a floral scent that is strongly dominated by *trans*-β-ocimene, its main site of emission are the petaloid lobes located at the flower limb, and its emissions describe an evening maximum between 17:00 and 20:00 that matches nicely with the flower opening and the activity of its crepuscular hawkmoth pollinators [36]. The floral scent of snapdragon (*Antirrhinum majus*) is dominated by *trans*-β-ocimene, whose emission follows a diurnal cycle controlled by a circadian clock, with the highest emission rates between 11:00 and 18:00 [16]. Dudareva et al. [16] showed that the rhythmic floral emission of *trans*-β-ocimene and other monoterpenes displayed a “free-running” cycle in the absence of environmental cues, with complete independence from light, indicating the circadian nature of its diurnal rhythmicity. The rates of emission of *trans*-β-ocimene from the flowers of snapdragon and Satsuma mandarin (*Citrus unshiu*) vary with floral ontogeny, with maximum emissions when flowers are fully open and mature and need to be pollinated, and decreasing emissions in later periods until fruit development [16,43]. Such spatial and temporal patterns of emission indicate a clear involvement of β-ocimene in the attraction of pollinators to flowers in these species [30]. However, the effects of β-ocimene on pollinators may be dependent on the context, i.e., on whether it is presented within the correct blend of floral and whole plant volatiles [44], and also with the correct combination of floral and plant traits other than scent.

Different patterns of emission are shown when floral volatiles develop biological functions other than pollinator attraction. For example, the emission of floral volatiles that serve as a defence against herbivores and florivores can be temporally induced after suffering an attack [45,46]; but constitutive floral emissions of these compounds that might interfere with pollination and constitute an unnecessary cost for the plant are generally avoided by animal-pollinated flowers [30,47,48]. Also, some volatile and non-volatile secondary metabolites with antibacterial and antifungal properties that develop defence functions against microbial pathogens are abundant in flowers [49,50]; they are constitutively produced and accumulated especially in floral tissues that the plant must protect even at a high cost because they are costly to produce, they are rich in nutrients, and they are important for plant reproduction, like for example nectar [51,52,53] and pollen [54,55]. Floral emissions of β-ocimene do not seem to play such roles as far as we read from the literature. Anyway, sometimes single floral volatiles can play multiple roles in flowers (e.g., flower defence and pollinator attraction) [50], and while the correct development of functions such as pollinator attraction or communication with other plant mutualists like predators of herbivores may be dependent on the correct combination with other compounds, some defensive functions might only depend on the presence of single compounds whose effectiveness is only dependent on their own deterrent, toxic [56,57] and antimicrobial properties [58,59] (even if combinations of defensive compounds can potentially show synergistic effects).

## 4. Biological Functions of β-Ocimene Emissions from Non-Floral Tissues

β-Ocimene is not only abundantly emitted by plant reproductive structures but is also a common VOC emitted from vegetative plant tissues [60,61]. Phytophagous insects can identify the VOC blends that are constitutively emitted by the plants in the community, including β-ocimene, and use them as chemical cues to identify their host plants [62,63].

β-Ocimene serves as a chemical cue in several plant species to attract natural enemies of phytophagous insects. Herbivore-infested plants induce increased emissions of VOCs such as β-ocimene from damaged and undamaged tissues in a systemic defensive response [64,65,66,67,68]. The emission of β-ocimene can also be induced in undamaged neighboring plants via volatile signals such as *cis*-jasmone from herbivore-attacked plants [68,69]. Parasitoids and predators of herbivores are attracted to the VOCs emitted from infested plants, which in most cases include important proportions of β-ocimene, that indirectly help plants to cope with herbivorous attacks in a tritrophic interaction [70,71]. The stronger production and emission of β-ocimene from herbivore-attacked *Medicago truncatula* plants compared to undamaged plants suggest that this compound plays an active role in indirect insect defenses [17]. In addition to its effect on insects, β-ocimene can also play important roles in plant-plant communication by mediating the induction of genes involved in defense [72,73]. Arimura et al. [72,73] revealed that β-ocimene and other volatiles emitted by lima bean after spider mite attack elicit the expression of several genes involved in plant defense by activation of the jasmonic acid signalling pathway, enabling these plants to prepare defenses against the spider mites in advance.

## 5. Conclusions

β-Ocimene is one of the most ubiquitous volatiles in floral scents and we have strong evidence to think that it can play relevant biological roles in flowers. To our knowledge there is no behavioural demonstration that β-ocimene attracts pollinators, but the abundant and well supported assumptions presented here warrant behavioural studies. There is strong indication that β-ocimene can play very relevant roles in the attraction of several types of pollinators to the flowers of a diverse array of plants. β-Ocimene also plays important defensive roles in vegetative plant tissues by mediating tritrophic interactions with parasitoids and predators of herbivores. We thus aim to highlight the importance of β-ocimene in the establishment of very important biological interactions between plants and beneficial organisms. In summary, β-ocimene has multiple relevant functions in plants, which vary depending on the organ and the time of emission. In view of the presented lines of indirect evidence, we strongly encourage the inclusion of β-ocimene alone or in combination with other floral volatiles in coupled gas chromatography electroantennographic detection (GC-EAD) analyses and behavioural tests when conducting future studies in order to provide a solid experimental proof for the assumptions made here.
